# Advanced-Stage Anal Squamous Cell Carcinoma Detected During Colonoscopy in a Rural HIV-Positive Patient: A Case Illustrating Fragmentation of Care and Barriers to Screening

**DOI:** 10.7759/cureus.108883

**Published:** 2026-05-15

**Authors:** Maxwell I Trettin, Robert S Glickenberger

**Affiliations:** 1 Osteopathic Medicine, Philadelphia College of Osteopathic Medicine, Moultrie, USA; 2 Gastroenterology and Hepatology, GI Associates of Big Bend, Tallahassee, USA

**Keywords:** anal canal squamous cell carcinoma, care fragmentation, human immuno-deficiency virus (hiv), rural health disparities, colonoscopy

## Abstract

Squamous cell carcinoma (SCC) of the anal canal is an uncommon malignancy with increasing incidence, particularly among people living with human immunodeficiency virus (HIV). In rural populations, limited access to specialized screening and fragmented care contribute to delayed diagnosis and advanced-stage presentation. A 58-year-old male patient with a 25-year history of HIV presented with one month of persistent rectal pain, intermittent bleeding, and mass sensation. Physical examination revealed a 5 × 6 cm ulcerated lesion near the anal margin. The patient reported inconsistent adherence to antiretroviral therapy due to transportation barriers and had no prior anorectal screening. Colonoscopy was performed, during which rectal retroflexion revealed an irregular, ulcerated, exophytic lesion with friability and infiltrative features. Biopsies and subsequent examination under anesthesia confirmed anal SCC. Staging demonstrated regional lymph node involvement without distant metastases, consistent with stage IIIA (T3N1M0). The patient was referred to colorectal surgery, radiation oncology, and medical oncology for further treatment. Ultimately, this case highlights how transportation barriers, inconsistent care access, and reliance on multiple geographically separated providers contributed to delayed diagnosis. In resource-limited settings, the lack of high-resolution anoscopy restricts early detection. Although not a primary screening tool, colonoscopy with careful anorectal evaluation may allow for opportunistic detection of malignancy in high-risk individuals. This case underscores the need for improved care integration and expanded access to screening resources among rural communities.

## Introduction

Squamous cell carcinoma (SCC) of the anal canal is a relatively uncommon malignancy among the general population, representing approximately 0.5% of all newly diagnosed cancers. The incidence of anorectal cancer has been steadily increasing over recent decades, particularly among high-risk populations, such as those with human immunodeficiency virus (HIV), where persistent oncogenic human papillomavirus (HPV) is the primary etiologic driver in approximately 90% of anal SCCs. People living with HIV (PLWH) have a nearly 20-fold higher risk due to chronic immune dysregulation, even in the modern era of highly active antiretroviral therapy (HAART). Among PLWH, sustained undetectable viral load is synonymous with lower prevalence of anal canal abnormalities and cancer risk, in which HAART can be protective against anal cancer [1]. However, several studies have noted that there are rural-urban disparities in access to and adherence to HAART, often citing challenges with linkage to care, household income, educational level, and limited healthcare access [2-3]. As a result, HIV-positive individuals are widely recognized as a key population in whom earlier detection of anorectal lesions may be beneficial.

Early detection of anorectal lesions often relies on specialized screening modalities, such as high-resolution anoscopy, anal cytology, HPV testing, and digital anorectal examination [4]. However, these screening modalities are not widely available in rural and resource-limited settings, often resulting in potential diagnostic delays [5]. These disparities are further compounded by care fragmentation within rural healthcare systems. In many rural regions, the management of HIV infection, gastrointestinal evaluation, and oncologic care occurs across disconnected clinical environments [6]. This fragmentation is further exacerbated by the limited local availability of subspecialists, including colorectal surgeons, anorectal oncologists, and HIV infectious disease specialists [7-8]. As a result, these patients in rural settings often experience prolonged intervals along the care continuum, which may result in a higher likelihood of advanced-stage presentation at diagnosis. Colonoscopy is not routinely used as a primary screening tool for anal cancers, but may incidentally identify suspicious neoplastic lesions during evaluation for other gastrointestinal conditions. Careful evaluation of the anorectal region, including retroflexion when appropriate, may allow for this incidental detection in patients who otherwise lack access to specialized screening modalities [9]. We present a case of a stage IIIA anal SCC identified during colonoscopy in an HIV-positive patient in a rural setting. This case highlights not only the potential role of colonoscopy as a diagnostic tool but also the impact that health care fragmentation has on delayed diagnosis in high-risk populations.

## Case presentation

**Figure 1:**
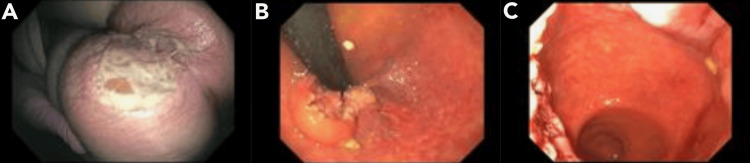
External and endoscopic views of a large, ulcerated anorectal mass. (A) Perianal lesion with irregular borders, central ulceration, and necrotic debris. (B) Colonoscopic visualization of a friable, exophytic mass within the anal canal upon retroflexion. (C) Colonoscopic visualization of luminal narrowing with surrounding mucosal erythema and distortion consistent with an advanced obstructive anorectal malignancy.

A 58-year-old man with a known history of HIV was referred to a rural gastroenterology practice for persistent rectal pain, intermittent rectal bleeding, and mass sensation for one month. Upon physical examination, a 5 × 6 cm ulcerated cutaneous lesion with centralized necrosis and purulent exudate was noted on the patient’s gluteal region, near the anal margin (Figure 1A). The lesion demonstrated irregular, poorly demarcated borders surrounded by erythema and induration. The patient had a 25-year history of HIV and was maintained on bictegravir-emtricitabine-tenofovir alafenamide (Biktarvy®). The patient’s HIV was primarily managed through primary care and intermittent infectious disease services. Most recent immunologic and virologic parameters before the procedure could not be attained. Past medical history was only notable for poor adherence to HAART and HIV maintenance, citing recurrent transportation issues. There was no known prior history of anorectal neoplasia, inflammatory bowel disease, HPV, or tobacco use disorder. Family history was noncontributory.

For further evaluation of the patient’s symptoms and presentation, a colonoscopy was performed under monitored anesthesia care without complication. During withdrawal from the cecum, careful inspection of the distal rectum and anorectal junction was performed. Upon retroflexion in the rectum, an irregular, ulcerated, exophytic lesion with friable mucosa was noted, demonstrating areas of mucosal breakdown (Figure 1B). The margins appeared poorly defined and infiltrative, with associated luminal narrowing and bleeding on contact (Figure 1C). Endoscopic appearance was concerning for malignancy, for which multiple biopsies were obtained using a cold biopsy technique. Hemostasis was achieved without complication, and no other lesions were identified. The patient subsequently underwent rectal examination under anesthesia (REUA) by a colorectal surgeon five weeks later. Histopathologic evaluation of the biopsy specimen, as documented in external records, utilized cytokeratin AE1/AE3 immunostaining and was reported as anal SCC in situ with features suspicious for superficial invasion. Following diagnosis, the patient underwent staging evaluation, including PET imaging, and was referred to medical and radiation oncology. According to external documentation, imaging demonstrated hypermetabolic right internal iliac and bilateral inguinal lymph nodes without evidence of distant metastases, and the disease was classified as stage IIIA (T3N1M0). The patient was subsequently managed with a multidisciplinary treatment approach, including concurrent 5-fluorouracil and mitomycin, interval anoscopy every 6-12 months, and planned surveillance imaging.

## Discussion

The present case highlights several multilevel care factors that likely contributed to the delayed recognition of advanced anal malignancy. This patient had a prolonged history of HIV with documented poor adherence to HAART, primarily attributed to transportation barriers and inconsistent access to care. This patient resided approximately 70 miles from their HIV specialist and 50 miles from the nearest gastroenterology and oncology practices. Transportation disparities are cited as primary contributors to poor treatment adherence among PLWH, particularly in rural regions. They are often associated with increased risk of persistent HPV infection and malignant sequelae [10]. In addition to geographic barriers, psychosocial factors, such as reduced self-efficacy, relationships with sexual partners, and stigma, may have further contributed to gaps in care reception. Behavioral constructs surrounding HIV and HPV have been shown to negatively influence care engagement and adherence [11]. While HIV viral load and CD4+ T-lymphocyte counts were unavailable at the time of presentation, the patient’s clinical and psychosocial history suggests periods of intermittent immunologic vulnerability.

This case underscores the significant fragmentation of care that is all too common within rural healthcare settings. The patient’s pre-diagnosis management was already distributed across multiple clinical settings, and their experienced transportation barriers only exaggerated this fragmentation when undergoing endoscopic and surgical evaluation. Distribution of care introduces multiple transitional points along the continuum, which are prone to delays in rural oncologic care [12]. Delays and fragmentation of care are consequential in the evaluation of suspected anorectal malignancy, in which there must be timely coordination among gastroenterology, colorectal surgery, pathology, oncology, and radiology for accurate diagnosis, staging, and initiation of treatment. In resource-limited settings, the absence of care integration and limited access to certain subspecialties may prolong time to diagnosis and delay initiation of oncologic therapy [13]. As demonstrated in this case, systemic barriers contributed to presentation at an advanced stage of disease, despite the presence of clinical symptoms in a high-risk individual. While colonoscopy is not a primary screening modality for anal SCC, this case highlights its vital role for opportunistic diagnoses, particularly in rural health settings where high-resolution anoscopy is limited. Careful considerations should be made by endoscopists for anorectal evaluation in high-risk individuals who lack access to specialized screening modalities. Nonetheless, reliance on incidental detection underscores a broader gap in preventive care infrastructure and reinforces the need for expanded access to targeted screening strategies among vulnerable populations.

## Conclusions

This case highlights the detection of stage IIIA anal SCC during colonoscopy in an HIV-positive patient residing in a rural setting. While colonoscopy is not the primary screening modality for anal malignancy, careful considerations by endoscopists for anorectal evaluation may facilitate opportunistic identification of suspicious lesions in high-risk individuals who lack access to specialized screening. This case underscores the impact of fragmented care and limited subspecialty access on delayed diagnosis and initiation of oncologic treatment. Even relatively short geographic distances may function as meaningful barriers to care in rural populations, especially when compounded by transportation limitations and the need for repeated, multidisciplinary visits such as those seen within oncology and HIV care.
